# Slow-4 cerebellar activity tracks MMSE language-related task performance across the Alzheimer’s disease spectrum: a multi-metric resting-state fMRI study

**DOI:** 10.3389/fnins.2026.1820150

**Published:** 2026-08-28

**Authors:** Haoran Mao, Bingqi Fu, Jiaying Song, Xize Jia, Lulu Cheng, He Xiao, Lianpeng Zhang

**Affiliations:** 1 School of Foreign Studies, China University of Petroleum (East China), Qingdao, China; 2 School of Information and Electronics Technology, Jiamusi University, Jiamusi, China; 3 School of Psychology, Zhejiang Normal University, Jinhua, China

**Keywords:** Alzheimer’s disease spectrum, cerebellar lobule VIII, frequency-specific spontaneous activity, MMSE language-related task performance, resting-state fMRI

## Abstract

**Background:**

Performance on MMSE language-related tasks may decline across the Alzheimer’s disease (AD) spectrum and has clinical relevance for characterizing cognitive change. However, because the MMSE language subscore is a brief composite screening measure rather than a domain-specific language battery, its neural correlates should be interpreted cautiously. The intrinsic neural activity patterns associated with MMSE language-related task performance across the AD continuum remain insufficiently characterized. Most prior studies have examined isolated functional metrics or focused on global cognition, leaving unclear whether multidimensional spontaneous activity measures and their frequency-specific properties converge on common neural substrates associated with this composite behavioral measure.

**Methods:**

We analyzed resting-state fMRI data from 175 ADNI participants (32 AD, 90 MCI, 53 CN), computing four complementary amplitude-based metrics—ALFF, fALFF, PerAF, and wavelet-ALFF—with wavelet-ALFF further decomposed into slow-4 (0.027–0.073 Hz) and slow-5 (0.01–0.027 Hz) bands. To examine functional alterations associated with MMSE language-related task performance, participants were grouped according to MMSE language sub-scores into intact performance, mild language-related impairment, and greater reduction in performance. Voxel-wise ANCOVA, partial correlation, and multiple linear regression analyses were performed to evaluate associations between resting-state metrics and MMSE language-related task performance, modeled both categorically and continuously, while controlling for age, sex, and education. Multiple comparisons were corrected using Gaussian Random Field theory.

**Results:**

Across diagnostic groups, amplitude-based metrics revealed frequency-dependent alterations: slow-5 band changes predominated in temporal regions, while slow-4 band alterations characterized parietal areas, particularly the right angular gyrus (127 voxels, peak *F* = 24.44). When stratified by MMSE language-related task performance, the paravermal (predominantly right) cerebellar lobule VIII emerged as the only region consistently identified across multiple metrics, including ALFF, PerAF, wavelet-ALFF, and across analytic approaches. Critically, the cerebellar lobule VIII finding showed its most consistent cross-analytic support in the slow-4 band. In ROI-based partial-correlation analyses controlling for age, sex, and education, higher slow-4 wavelet-ALFF in this region was negatively associated with MMSE language-related task performance, and this association remained significant after additional adjustment for global disease severity (CDR-SB; partial *r* = −0.31, *p* < 0.001) and for non-language MMSE performance (partial *r* = −0.31, *p* < 0.001). The negative direction—whereby greater activity in this cerebellar cluster accompanied poorer performance—was consistent across conventional broadband, slow-4, and slow-5 analyses.

**Conclusion:**

These findings suggest that right cerebellar lobule VIII activity is meaningfully associated with performance on MMSE language-related tasks across the AD spectrum, particularly within the slow-4 frequency range. Importantly, because the MMSE language subscore includes naming, repetition, reading, writing, command execution, and constructional praxis, the observed associations should not be interpreted as evidence for a purely language-specific cerebellar mechanism. Rather, they may reflect a combination of linguistic, executive, visuospatial, motor-planning, and sensorimotor processes required by MMSE language-related tasks. More broadly, the present study highlights the value of frequency-resolved spontaneous activity analyses for identifying clinically relevant brain-behavior associations in AD and suggests that cerebellar circuits warrant further investigation in relation to composite cognitive and language-related task performance.

## 1 Introduction

Alzheimer’s disease (AD) is no longer conceptualized as a binary diagnostic entity but rather as a biological continuum spanning from cognitively normal aging through mild cognitive impairment (MCI) to overt dementia (Molinuevo et al., 2010; Jack et al., 2013). This fundamental paradigm shift has profound implications for understanding how specific cognitive domains—particularly language—deteriorate as neuropathology progressively accumulates across the disease spectrum. Although episodic memory deficits remain the clinical hallmark of AD, reduced performance on MMSE language-related tasks is increasingly recognized as an early and functionally meaningful feature of the disease (Verma and Howard, 2012; Leavitt et al., 2026). The MCI stage represents a critical transitional window during which functional alterations may precede irreversible structural degeneration, underscoring the urgency of characterizing neurobiological changes across the CN-MCI-AD continuum before extensive neuronal loss precludes effective intervention.

The clinical significance of reduced performance on MMSE language-related tasks in AD extends far beyond its role as a diagnostic marker. Patients and families consistently rank communication difficulties among the most distressing aspects of the disease, profoundly affecting social engagement, functional independence, and quality of life (Xiao et al., 2025). Recent work has further suggested that language-related decline may not merely be a downstream consequence of global cognitive deterioration but could itself contribute to it by disrupting verbally mediated processes (Leavitt et al., 2026), a possibility that, if confirmed, would heighten the importance of characterizing its neural correlates with greater precision. Within large-scale datasets such as the Alzheimer’s Disease Neuroimaging Initiative (ADNI), the Mini-Mental State Examination (MMSE) language sub-scale provides a standardized clinical measure to quantify these changes (Jahromi et al., 2024), enabling systematic investigation of brain–behavior relationships across the continuum.

Neuroimaging studies have made significant strides in mapping the neural correlates of performance on language-related tasks in AD. Early lesion and structural imaging work implicated left-hemisphere temporoparietal regions, establishing that language deficits in AD are not random but reflect selective network vulnerability (Vuilleumier, 2013). More recently, task-based and resting-state functional connectivity studies have extended these findings. Xiao et al. (2025) demonstrated that fronto-temporal functional connectivity mediates the relationship between amyloid-β burden and language performance, suggesting a pathway from molecular pathology to clinical symptoms. Complementing this connectivity work, Huang et al. (2024) identified temporo-frontoparietal hypoconnectivity as a potential biomarker for isolated reduced performance on language-related tasks in MCI, with particular involvement of the superior temporal pole and supramarginal gyrus. Collectively, these findings converge on a network-level account of variation in language-related task performance, implicating distributed cortical systems rather than isolated regions.

Yet three critical gaps remain that limit our mechanistic understanding of the neural basis of MMSE language-related task performance in AD. First, the vast majority of studies have examined functional connectivity between regions, leaving unanswered whether local intrinsic activity—the “baseline” neural dynamics within individual regions—is altered in ways that contribute to individual differences in performance on language-related tasks. This distinction matters fundamentally because connectivity changes could arise from disrupted local signal generation, altered inter-regional coupling, or both. Without characterizing local activity, we cannot determine whether connectivity alterations reflect primary regional dysfunction or secondary compensatory processes. Second, despite growing recognition that neural oscillations are frequency-specific, most resting-state fMRI studies collapse across frequency bands using conventional ALFF or fALFF measures. Emerging evidence suggests that slow-4 (0.027–0.073 Hz) and slow-5 (0.01–0.027 Hz) oscillations may carry distinct physiological signatures and disease-related information (Zuo et al., 2010; Yue et al., 2023). Slow-5 oscillations have been linked to cortical slow waves and default-mode network integration, while slow-4 oscillations are more closely associated with cortico-subcortical information transfer. However, this frequency specificity has not been systematically leveraged in the study of AD-related language decline. Third, and most importantly, the cerebellum has been conspicuously absent from the conversation. While traditionally overlooked in AD research—viewed as a motor structure relatively spared by AD pathology—the cerebellum is increasingly recognized for its role in higher-order cognition and linguistic processing (Schmahmann, 2019; Guell et al., 2018). Cerebro-cerebellar closed-loop circuits connect the cerebellum with prefrontal, posterior parietal, and temporal cortices, providing an anatomical substrate for cerebellar participation in semantic, phonological, and syntactic operations. Whether cerebellar intrinsic activity is altered across the AD spectrum and whether such alterations track with language performance remains entirely unknown.

Resting-state fMRI offers a window into intrinsic brain activity without task-related confounds, making it particularly suitable for studying clinical populations across the disease continuum. Multiple complementary metrics have been developed to capture different facets of local neural dynamics. The amplitude of low-frequency fluctuation (ALFF) and its normalized form fractional ALFF (fALFF) index the intensity of spontaneous oscillations (Zang et al., 2007; Zou et al., 2008). Percent amplitude of fluctuation (PerAF) provides a standardized measure of signal variability with improved test–retest reliability across multi-center datasets such as ADNI (Jia et al., 2020; Hammers et al., 2022). More recently, wavelet-based approaches have enabled decomposition of BOLD signals into distinct frequency bands, revealing that slow-4 and slow-5 oscillations may have differential sensitivity to neurodegenerative pathology (Yue et al., 2023). Each metric captures a different aspect of neural function, yet prior studies have typically employed them in isolation or focused on global cognition. A multi-metric integration framework—one that asks how these indices converge, diverge, and differentially relate to language performance across the AD continuum—has been conspicuously lacking.

The present study was designed to address these gaps by asking three specific questions: First, do local spontaneous neural activity metrics—ALFF, fALFF, PerAF, and wavelet-ALFF—differ across the AD-MCI-CN spectrum in ways that are detectable and reproducible? Second, are these metric alterations frequency-dependent, with slow-4 and slow-5 bands showing distinct patterns of group differences? Third, and most critically, which of these metric alterations correlate with language performance, and do these correlations converge on a common set of brain regions?

Based on prior literature, we hypothesized that lower MMSE language-related task performance would be associated with convergent alterations across multiple metrics in regions traditionally implicated in language—left-lateralized temporoparietal and frontal areas. However, we also remained open to the possibility that frequency-resolved, multi-metric analysis might reveal unexpected patterns. Indeed, the fact that the cerebellum consistently appeared across metrics and frequency bands became the study’s most provocative and unanticipated discovery, challenging the prevailing cortical-centric view of AD-related language decline. Identifying such multi-metric functional signatures may advance mechanistic understanding of language vulnerability in AD and contribute to the development of clinically informative neuroimaging biomarkers for monitoring disease progression and evaluating therapeutic interventions.

## 2 Materials and methods

### 2.1 Participants

Data used in the preparation of this article were obtained from the Alzheimer’s Disease Neuroimaging Initiative (ADNI) database (adni.loni.usc.edu). The ADNI was launched in 2003 as a public-private partnership, led by Principal Investigator Michael W. Weiner, MD. The primary goal of ADNI has been to test whether serial magnetic resonance imaging (MRI), positron emission tomography (PET), other biological markers, and clinical and neuropsychological assessment can be combined to measure the progression of mild cognitive impairment (MCI) and early Alzheimer’s disease (AD).

Inclusion required a high-resolution T1-weighted MPRAGE and a resting-state fMRI scan from the same ADNI visit. For participants with longitudinal data, the earliest qualifying visit was selected to maximize sample size while avoiding practice effects.

Diagnostic grouping followed the standard ADNI criteria. Notably, to capture the full clinical spectrum of prodromal Alzheimer’s disease and maximize statistical power, participants initially classified as Early MCI (EMCI) and Late MCI (LMCI) were merged into a single MCI group. This consolidation is justified as EMCI and LMCI represent different stages along the same continuous cognitive decline trajectory (Petersen et al., 2010; Jack et al., 2013). Consequently, participants were classified into cognitively normal (CN), mild cognitive impairment (MCI), and Alzheimer’s disease (AD) groups. All included participants completed the Mini-Mental State Examination (MMSE). To index language performance, language-related sub-item scores from the MMSE (e.g., naming, repetition, reading, and writing) were extracted and used as primary clinical measures of linguistic function.

Applying the above inclusion criteria yielded an initial sample of 196 participants: 34 individuals with AD, 105 with MCI (including both EMCI and LMCI), and 57 CN controls. The CN group was frequency-matched to the patient groups with respect to age, sex, and years of education. After applying head motion (translation > 3 mm or rotation >3°) and data quality exclusions, the final sample comprised 175 participants: 32 with AD, 90 with MCI, and 53 CN controls.

### 2.2 Clinical and language assessment

Language performance was quantified using the MMSE language sub-score (range 0–9), which includes naming, repetition, three-step command, reading, writing, and constructional praxis. While the MMSE is not a comprehensive language battery, its standardized administration across the ADNI cohort enables cross-sectional comparisons that would be impossible with heterogeneous clinical assessments. This trade-off—depth versus standardization—is inherent to multi-center database research and must be acknowledged.

Participants were stratified into three language groups based on sub-score distribution: intact (9/9), mild impairment (7–8), and moderate-to-severe impairment (0–6). These groupings were intended to balance interpretability with adequate statistical power for group comparisons rather than to represent formally validated clinical language severity classifications. The rationale for these cutoffs was based on the distributional characteristics of the MMSE language sub-score and the nature of its component tasks. Because the MMSE language items assess relatively fundamental functions, cognitively intact older adults typically demonstrate ceiling-level performance (score = 9). Scores of 7–8 generally reflect isolated errors on one or more items (e.g., naming or complex repetition), whereas scores ≤ 6 indicate broader impairment across multiple language-related tasks within the context of this brief screening measure. Importantly, because the MMSE language sub-score combines partially overlapping domains—including naming, repetition, reading, writing, and command following—the present groupings should be interpreted cautiously and not as reflecting isolated linguistic subdomains.

### 2.3 Data acquisition

All neuroimaging data were acquired at multiple ADNI sites using 3.0 Tesla MRI systems from different manufacturers (Siemens and Philips). Acquisition protocols followed standardized ADNI procedures to ensure cross-site harmonization.

#### 2.3.1 Structural MRI

High-resolution T1-weighted structural images were obtained using a three-dimensional Magnetization-Prepared Rapid Gradient Echo (3D MPRAGE) sequence. Typical acquisition parameters included: repetition time (TR) = 2,300 ms, echo time (TE) = 3.0 ms, inversion time (TI) = 900 ms, flip angle = 9°, slice thickness = 1.2 mm, and in-plane resolution of 1.0 × 1.0 mm^2^ (matrix size = 240 × 256). These parameters were optimized to facilitate accurate spatial normalization and anatomical segmentation.

#### 2.3.2 Resting-state fMRI

Resting-state functional MRI (rs-fMRI) data were acquired using gradient-echo echo-planar imaging (EPI) sequences. Typical acquisition parameters included: TR = 3,001 ms, TE = 30 ms, flip angle = 80°, and isotropic voxel size of 3.3 × 3.3 × 3.3 mm^3^.

During rs-fMRI acquisition, participants were instructed to remain still and relaxed. Depending on the specific ADNI sub-protocol, scans were conducted with eyes open or eyes closed. No explicit cognitive task was administered during scanning.

### 2.4 Data preprocessing

Preprocessing was performed using RESTplus V1.25 (Jia et al., 2019) and SPM12. The first 10 volumes were discarded to allow for magnetic field stabilization and participant adaptation. The remaining images underwent slice-timing correction, head-motion correction (participants with head motion exceeding 3 mm displacement or 3° rotation were excluded), spatial normalization to the Montreal Neurological Institute (MNI) space (3 × 3 × 3 mm^3^ resampling), and spatial smoothing using a 6-mm full-width at half-maximum (FWHM) Gaussian kernel. Linear detrending and nuisance regression using the Friston-24 head motion parameters were subsequently performed.

Different preprocessing strategies were adopted according to the characteristics of each rs-fMRI metric. For ALFF, fALFF, and Wavelet-ALFF calculations, the detrended and nuisance-regressed time series without temporal band-pass filtering were used. This approach was adopted because these metrics intrinsically characterize spontaneous BOLD fluctuations through frequency-domain decomposition or wavelet transformation, and additional filtering may alter the original frequency distribution of the signal.

For PerAF calculation, an additional temporal band-pass filtering step (0.01–0.08 Hz) was applied to the residual time series before amplitude estimation. This filtering procedure was applied to attenuate very-low-frequency drift and high-frequency physiological fluctuations outside the frequency range of interest. Therefore, PerAF was calculated from the band-pass filtered time series, whereas ALFF, fALFF, and Wavelet-ALFF were calculated from the unfiltered residual time series.

### 2.5 Calculation of rs-fMRI metrics

To comprehensively characterize spontaneous local neural dynamics, four complementary resting-state fMRI metrics were computed, including ALFF, fALFF, PerAF, and Wavelet-ALFF. All rs-fMRI metrics were calculated using metric-specific preprocessed time series before statistical inference. ALFF, fALFF, and Wavelet-ALFF were derived from the detrended and nuisance-regressed time series without temporal filtering, whereas PerAF was calculated from the additionally band-pass filtered (0.01–0.08 Hz) time series. The different preprocessing streams were selected based on the computational characteristics of each metric. To localize regions exhibiting significant functional alterations, we utilized the Automated Anatomical Labeling atlas (AAL; Tzourio-Mazoyer et al., 2002), a standardized anatomical parcellation framework that divides the brain into 116 cortical and subcortical regions, including cerebellar subdivisions.

#### 2.5.1 ALFF, fALFF, and PerAF

The Amplitude of Low-Frequency Fluctuation (ALFF) was computed by transforming the detrended and nuisance-regressed voxel-wise time series into the frequency domain using a Fast Fourier Transform (FFT). The power spectrum was obtained, and the square root of the power within the low-frequency band (0.01–0.08 Hz) was averaged to yield the ALFF value for each voxel. This frequency range was selected based on previous evidence suggesting that low-frequency oscillations in this band primarily reflect spontaneous neural activity. No prior temporal band-pass filtering was applied because ALFF calculation directly extracts the power of low-frequency oscillations (0.01–0.08 Hz) from the frequency spectrum.

Fractional ALFF (fALFF) was calculated using the unfiltered residual time series as the ratio of power within the low-frequency band (0.01–0.08 Hz) to the total power across the entire measurable frequency spectrum. This normalization procedure reduces the contribution of non-specific fluctuations and may improve the specificity of spontaneous neural activity measurements.

PerAF was computed as the percentage fluctuation of the BOLD signal relative to its mean intensity at each voxel. The calculation was performed using the band-pass filtered (0.01–0.08 Hz) time series described above. Compared with conventional amplitude-based measures, PerAF provides a scale-independent measure of BOLD signal variability and has demonstrated favorable inter-subject comparability.

To minimize inter-individual global signal variability, voxel-wise ALFF, fALFF, and PerAF maps were standardized by dividing each voxel value by the global mean within the brain mask and subsequently converted to z-scores for group-level analyses.

#### 2.5.2 Wavelet-ALFF

Wavelet-ALFF was computed using a discrete wavelet transform with a Daubechies 4 basis applied to the detrended and nuisance-regressed BOLD time series. Following Zuo et al. (2010), we examined slow-5 (0.01–0.027 Hz; wavelet scale 4) and slow-4 (0.027–0.073 Hz; wavelet scale 3). These bands were selected because prior work has demonstrated distinct spatial distributions and reliability profiles for slow-4 and slow-5 resting-state BOLD fluctuations. No prior temporal band-pass filtering was performed because wavelet decomposition itself separates the signal into predefined frequency components.

### 2.6 Statistical analysis

Voxel-wise group comparisons were performed using one-way ANCOVA in DPABI/SPM12, with age, sex, education included as covariates across all group-level analyses. Multiple comparisons were corrected using Gaussian Random Field (GRF) theory. Because ALFF, fALFF, PerAF, and wavelet-ALFF differ systematically in their smoothness and signal-to-noise characteristics, a single voxel-level threshold can over- or under-correct depending on the metric (Chen et al., 2018). We therefore report results at a uniform cluster-level threshold of *p* < 0.05 (two-tailed, GRF-corrected) while specifying the voxel-level threshold for each metric in the corresponding tables. Critically, to guard against threshold-dependent inflation of the central cerebellar finding, we verified that the right (paravermal) lobule VIII cluster survived the most stringent voxel-level threshold (*p* < 0.001) in both the slow-4 categorical ANCOVA and the slow-4 continuous regression. The convergence of the cerebellar finding under this conservative threshold, and across categorical and continuous analyses, indicates that it is not an artifact of liberal voxel-level thresholding.

Voxel-wise multiple linear regression analyses were performed to examine associations between each rs-fMRI metric and MMSE language-related task performance, with age, sex, and education included as covariates. In each model, the voxel-wise rs-fMRI metric was entered as the dependent variable and the MMSE language subscore as the predictor of interest. The resulting *t-values* are reported for the voxel-wise analyses, with metric-specific GRF-corrected thresholds. Partial correlation analyses were subsequently conducted for the ROI-based sensitivity analyses, for which partial r values are reported.

To address the possibility that associations between cerebellar activity and MMSE language-related task performance might reflect global cognitive impairment or overall disease severity, we performed additional ROI-based sensitivity analyses. Mean slow-4 wavelet-ALFF values were extracted from the paravermal (predominantly right) lobule VIII cluster identified in the main analysis. Partial correlations were then conducted between the extracted ROI values and MMSE language subscores.

**FIGURE 1:**
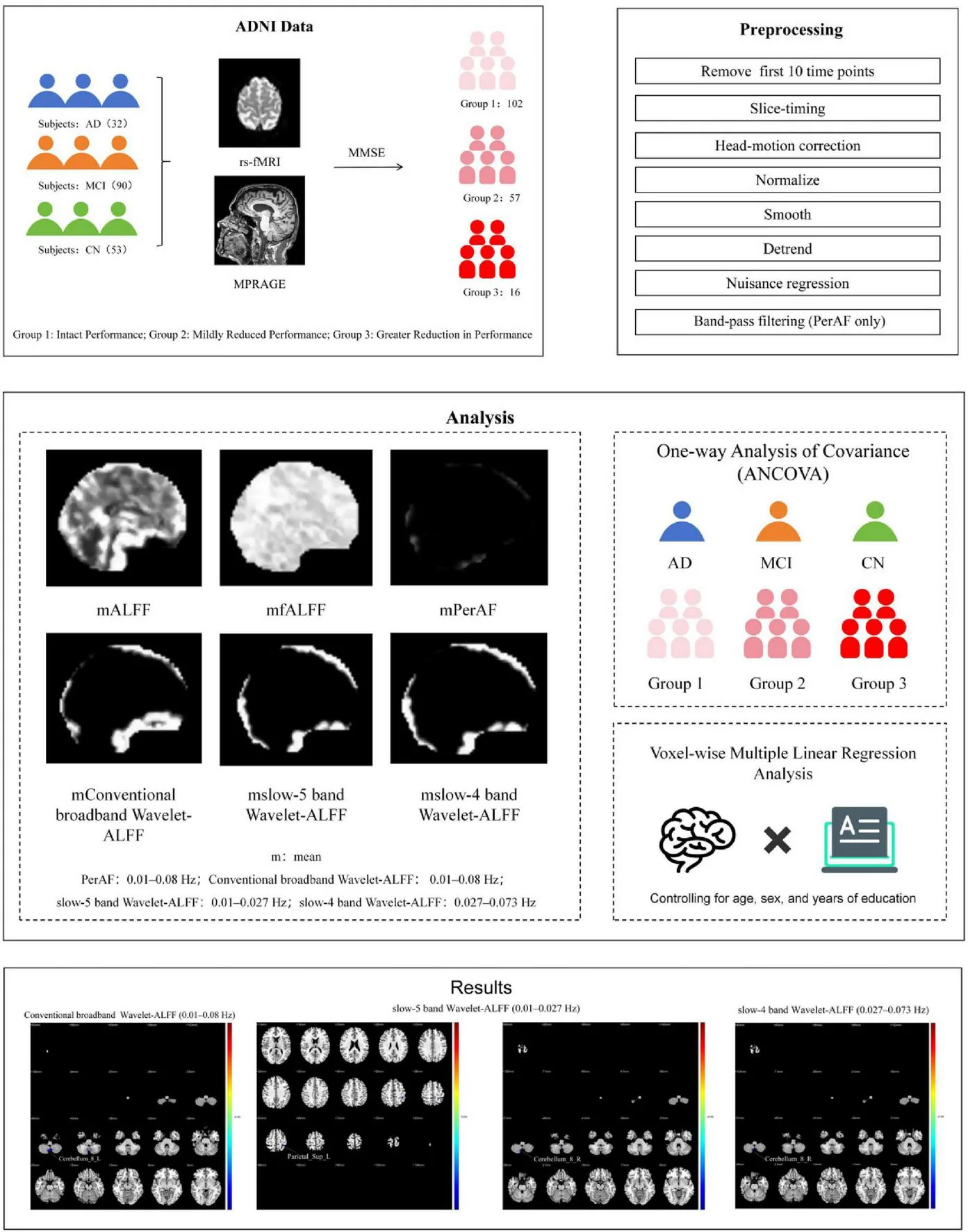
Overall workflow.

Because the MMSE total score includes the MMSE language subscore, directly controlling for MMSE total score would introduce construct overlap. We therefore used two complementary adjustment models. In Model A, age, sex, education, and CDR-SB were included as covariates to account for global clinical disease severity. In Model B, age, sex, education, and the MMSE non-language score were included as covariates. The MMSE non-language score was calculated by subtracting the MMSE language subscore from the MMSE total score. The overall preprocessing and analysis workflow is shown in Figure 1.

## 3 Results

### 3.1 Demographic and clinical characteristics

Due to head motion and data quality issues, we excluded 2 individuals with AD, 15 individuals with MCI (including both EMCI and LMCI), and 4 CN controls. The final sample consisted of 175 participants: 32 with AD, 90 with MCI, and 53 CN controls (Table 1). No significant age differences emerged across diagnostic groups [*F*(2, 172) = 2.90, *p* = 0.057; Table 2], although CN participants were slightly older (75.1 ± 6.8 years) than AD (72.2 ± 7.2 years) and MCI (72.3 ± 7.2 years) participants (Table 1). *Post-hoc* tests confirmed that all pairwise comparisons were non-significant (*p* > 0.05). Age was therefore included as a covariate in all subsequent imaging analyses.

**TABLE 1:** Participant demographics and age distribution by groups.

Group	n	Mean age (years) ± SD	Male	Female
AD	32	72.22 ± 7.18	15	17
CN	53	75.06 ± 6.83	21	32
MCI	90	72.25 ± 7.24	45	45
Total	175	73.09 ± 7.19	81	94

**TABLE 2:** One-way ANOVA results for age across diagnostic groups.

Variable	AD (*N* = 32)		CN (*N* = 53)		MCI (*N* = 90)		ANOVA results	*Post hoc* (Tukey)
	M	SD	M	SD	M	SD		
Age	72.22	7.18	75.06	6.83	72.25	7.24	*F*(2, 172) = 2.90	n.s.

Based on MMSE language subscores, participants were stratified into three performance groups: intact performance (score = 9, *n* = 102), mildly reduced performance (scores = 7–8, *n* = 57), and greater reduction in performance (scores ≤ 6, *n* = 16). Demographic characteristics of these groups are presented in Table 3. MMSE language-related task scores were associated with sex and education, but not with age (Table 4).

**TABLE 3:** Participant demographics and age distribution by MMSE language-related task performance groups.

Group	N	Mean age (years) ± SD	Male	Female
Intact performance	102	72.75 ± 7.23	40	62
Mildly reduced performance	57	73.32 ± 6.61	33	24
Greater reduction in performance	16	74.47 ± 8.55	8	8
Total	175	73.09 ± 7.19	81	94

**TABLE 4:** Associations of MMSE language-related task scores with age, education, and sex.

Variable pair	Statistical test	Test statistic	*p*-value
MMSE language related task score vs. age	Spearman’s rank correlation	−0.08	0.31
MMSE language related task score vs. education	Spearman’s rank correlation	0.16	0.03*
MMSE language related task score by sex	Mann-Whitney U test	3216	0.04*

*p < 0.05.

### 3.2 Group differences in rs-fMRI metrics among AD, MCI, and CN

#### 3.2.1 ALFF, fALFF, and PerAF

To minimize the impact of inter-subject variability and enhance the reliability of group comparisons, all voxel-wise metrics, including ALFF, fALFF, and PerAF, were standardized by dividing the value of each voxel by the global mean within the whole-brain mask (yielding mALFF, mfALFF, and mPerAF, respectively). This mean-standardization procedure is a widely adopted approach in resting-state fMRI studies to reduce global variance and improve the sensitivity of clinical findings (Zuo et al., 2010; Yan et al., 2013).

Subsequently, a one-way analysis of covariance (ANCOVA) was performed to identify brain regions showing significant differences in these metrics among the AD, MCI, and CN groups, with age, sex, and years of education included as covariates systematically across all models. The resulting statistical maps were corrected for multiple comparisons using Gaussian Random Field (GRF) theory (voxel-level *p* < 0.001, cluster-level *p* < 0.05, two-tailed), ensuring rigorous control of false-positive results.

As summarized in Table 5 and illustrated in Supplementary Figures S1–S3, the one-way ANCOVA revealed significant group differences across several brain regions for all three amplitude-based metrics. *Post-hoc* analyses (Bonferroni corrected) further elucidated the specific patterns of these inter-group differences.

**TABLE 5:** Brain regions showing significant differences in ALFF, fALFF, and PerAF among AD, MCI, and CN groups (ANCOVA).

			MNI coordinate (mm)			
Metric/regions	Cluster size	Peak *F*-value	*X*	*Y*	*Z*	*Post hoc*
ALFF						
SupraMarginal_R	17	10.94	69	−21	18	AD > CN, AD > MCI
fALFF						
Temporal_Mid_R	9	10.74	63	−33	6	AD > MCI, CN > MCI
Temporal_Mid_R	28	15.18	42	−66	0	AD > MCI, CN > MCI
Postcentral_L	11	14.68	−54	−6	42	AD > MCI, CN > MCI
Frontal_Mid_R	54	13.83	36	12	57	AD > MCI, CN > MCI
Precentral_L	61	13.35	−36	−12	63	AD > MCI, CN > MCI
Frontal_Mid_L	11	11.98	−39	9	48	AD > MCI, CN > AD, CN > MCI
Frontal_Sup_R	37	16.08	24	−3	72	AD > MCI, CN > MCI
PerAF						
Temporal_Inf_L	41	19.62	−54	−6	−33	AD > CN, AD > MCI

AD, Alzheimer’s disease; MCI, mild cognitive impairment; CN, cognitively normal; ALFF, amplitude of low-frequency fluctuation; fALFF, fractional ALFF; PerAF, percent amplitude of fluctuation; MNI, Montreal Neurological Institute. All results were corrected using Gaussian Random Field (GRF) theory (voxel-level *p* < 0.001, cluster-level *p* < 0.05, two-tailed).

Specifically, for mALFF, a significant cluster was identified in the right supramarginal gyrus (Peak *F* = 10.94, Cluster size = 17 voxels). *Post-hoc* comparisons revealed that the AD group exhibited significantly higher mALFF values in this region compared to both the CN and MCI groups (AD > CN, AD > MCI).

For mfALFF, significant alterations were widely distributed across multiple regions. In the frontal lobe, differences were observed in the right superior frontal gyrus (Peak *F* = 16.08), right middle frontal gyrus (Peak *F* = 13.83), and left middle frontal gyrus (Peak *F* = 11.98). In the temporal lobe, two significant clusters were identified in the right middle temporal gyrus (Peak *F* = 15.18 and Peak *F* = 10.74). Additionally, alterations were noted in the left precentral gyrus (Peak *F* = 13.35) and left postcentral gyrus (Peak *F* = 14.68). Notably, *post-hoc* results for most of these regions (except the left middle frontal gyrus) followed a consistent pattern where the MCI group showed significantly reduced mfALFF compared to both AD and CN groups (AD > MCI, CN > MCI). In the left middle frontal gyrus, a progressive decrease was observed from the CN group to the AD group, and finally to the MCI group (CN > AD > MCI).

For mPerAF, significant differences were identified in the left inferior temporal gyrus (Peak *F* = 19.62, Cluster size = 41 voxels). *Post-hoc* analyses indicated that mPerAF values in this region were significantly elevated in the AD group relative to the CN and MCI groups (AD > CN, AD > MCI).

Overall, the different amplitude-based metrics demonstrated distinct sensitivities to regional functional alterations. While mALFF and mPerAF highlighted localized AD-specific hyperactivation (e.g., AD > CN, AD > MCI) in the right supramarginal gyrus and left inferior temporal gyrus, respectively, mfALFF appeared to be more sensitive to widespread functional changes across the frontal and temporo-sensorimotor regions, particularly highlighting a marked reduction of activity in the MCI stage.

#### 3.2.2 Frequency-specific alterations via wavelet-ALFF

To explore the frequency-specific patterns of neural oscillations, Wavelet-ALFF (W-ALFF) was analyzed across three distinct frequency bands: the conventional broadband (0.01–0.08 Hz), slow-5 band (0.01–0.027 Hz), and slow-4 band (0.027–0.073 Hz). One-way ANCOVA revealed significant group differences in W-ALFF across all three bands among the AD, MCI, and CN groups (Table 6 and Supplementary Figures S4–S6).

**TABLE 6:** Brain regions showing significant differences in frequency-specific alterations via wavelet-ALFF among AD, MCI, and CN groups (ANCOVA).

			MNI coordinate (mm)			
Frequency band/regions	Cluster size	Peak *F*-value	*X*	*Y*	*Z*	*Post hoc*
Conventional broadband wavelet-ALFF (0.01–0.08 Hz)						
SupraMarginal_R	19	11.44	69	−21	18	AD > CN, CN > MCI
Slow-5 band wavelet-ALFF (0.01–0.027 Hz)						
Temporal_Mid_L	17	12.08	−57	3	−27	AD > CN, AD > MCI
Temporal_Mid_L	12	11.66	−63	−48	0	AD > MCI, CN > MCI
Temporal_Mid_R	73	15.79	57	−63	15	AD > MCI, CN > MCI
SupraMarginal_L	19	18.77	−63	−42	30	AD > MCI, CN > MCI
Angular_R	52	23.10	57	−57	36	AD > MCI, CN > MCI
Slow-4 band wavelet-ALFF (0.027–0.073 Hz)						
Temporal_Mid_L	15	12.19	-57	3	-27	AD > CN, AD > MCI, CN > MCI
Temporal_Mid_R	25	15.36	63	0	−18	AD > CN, AD > MCI
Angular_R	127	24.44	57	−57	36	AD > MCI, CN > MCI
SupraMarginal_L	17	17.33	−63	−42	30	AD > MCI, CN > MCI

All results were corrected using Gaussian Random Field (GRF) theory (voxel-level *p* < 0.001, cluster-level *p* < 0.05, two-tailed).

Notably, the spatial distribution and extent of significant clusters exhibited clear frequency-dependent characteristics. In the conventional broadband, significant alterations were primarily localized in the right supramarginal gyrus (Peak *F* = 11.44, Cluster size = 19 voxels). *Post-hoc* analyses indicated that the AD group had higher values than the CN group, while the MCI group showed the lowest activity (AD > CN, CN > MCI).

The slow-5 and slow-4 bands demonstrated distinct sensitivities and more widespread alterations compared to the conventional band. In the slow-5 band, alterations were predominantly observed in the temporoparietal regions, including the bilateral middle temporal gyrus, left supramarginal gyrus, and right angular gyrus. *Post-hoc* analyses revealed a prevalent pattern of MCI-specific reduction in these regions (e.g., right middle temporal gyrus, left supramarginal gyrus, and right angular gyrus; AD > MCI, CN > MCI). Additionally, a sub-cluster in the left middle temporal gyrus showed significantly elevated slow-5 activity in the AD group compared to both CN and MCI groups (AD > CN, AD > MCI).

In the slow-4 band, alterations in the parietal regions remained prominent and extensive. Specifically, the right angular gyrus (Peak *F* = 24.44, Cluster size = 127 voxels) and left supramarginal gyrus showed significant group differences, characterized by a marked reduction in the MCI group (AD > MCI, CN > MCI). Furthermore, temporal regions also showed specific alterations: the left middle temporal gyrus exhibited a progressive pattern of alteration where the AD group showed the highest activity and the MCI group showed the lowest (AD > CN, AD > MCI, CN > MCI), while the right middle temporal gyrus showed elevated activity in the AD group (AD > CN, AD > MCI).

Overall, these findings suggest that the slow-4 band is particularly sensitive to extensive functional changes in major parietal hubs (e.g., the right angular gyrus), while both bands robustly capture alterations across temporal and parietal networks in the context of cognitive decline.

### 3.3 Brain functional alterations across levels of MMSE language-related task performance

#### 3.3.1 ALFF, fALFF, and PerAF

One-way ANCOVA was performed to identify brain regions showing significant differences in spontaneous neural activity among the three groups categorized according to MMSE language-related task performance (Intact Performance Group, Mildly Reduced Performance Group, and Greater Reduction in Performance Group). The results are summarized in Table 7. To balance sensitivity and specificity across different metrics, specific Gaussian Random Field (GRF) correction thresholds were applied for each metric.

**TABLE 7:** Brain regions showing significant differences in ALFF, fALFF, and PerAF among intact performance group, mildly reduced performance group, and greater reduction in performance group based on MMSE language-related task performance.

			MNI coordinate (mm)			
Metric/regions	Cluster size	Peak *F*-value	*X*	*Y*	*Z*	*Post hoc*
ALFF						
Cerebellum_8_R	119	10.49	9	−69	−48	Mildly reduced > Intact, Greater reduction > Intact
fALFF						
Occipital_Mid_L	3	12.36	−30	−81	27	Mildly reduced > Intact, Greater reduction > Intact
PerAF						
Cerebellum_8_R	58	10.70	9	−69	−48	Greater reduction > Intact, Greater reduction > Mildly reduced

All results were corrected using Gaussian Random Field (GRF) theory (ALFF: voxel-level *p* < 0.05, cluster-level *p* < 0.05, two-tailed; fALFF: voxel-level *p* < 0.001, cluster-level *p* < 0.05, two-tailed; PerAF: voxel-level *p* < 0.01, cluster-level *p* < 0.05, two-tailed).

For mALFF (thresholded at voxel-level *p* < 0.05, cluster-level *p* < 0.05), a significant cluster was identified in the paravermal (predominantly right) lobule VIII (labeled as Cerebellum_8_R in the AAL atlas) (Peak *F* = 10.49, Cluster size = 119 voxels). *Post-hoc* analyses revealed that both the Mildly Reduced Performance Group and Greater Reduction in Performance Group exhibited significantly higher mALFF values in this lobule VIII cluster compared to the Intact Performance Group. For mfALFF, thresholded at voxel-level *p* < 0.001 and cluster-level *p* < 0.05, a significant cluster was identified in the left middle occipital gyrus (peak *F* = 12.36; cluster size = 3 voxels). Post-hoc comparisons showed higher mfALFF values in both the mildly reduced performance and greater reduction in performance groups than in the intact performance group.

For mPerAF (thresholded at voxel-level *p* < 0.01, cluster-level *p* < 0.05), significant group differences were also identified in the right cerebellum 8 (Peak *F* = 10.70, Cluster size = 58 voxels). *Post-hoc* results showed that the greater reduction in performance group exhibited significantly higher mPerAF values than both the intact performance and mildly reduced performance groups.

**FIGURE 2:**
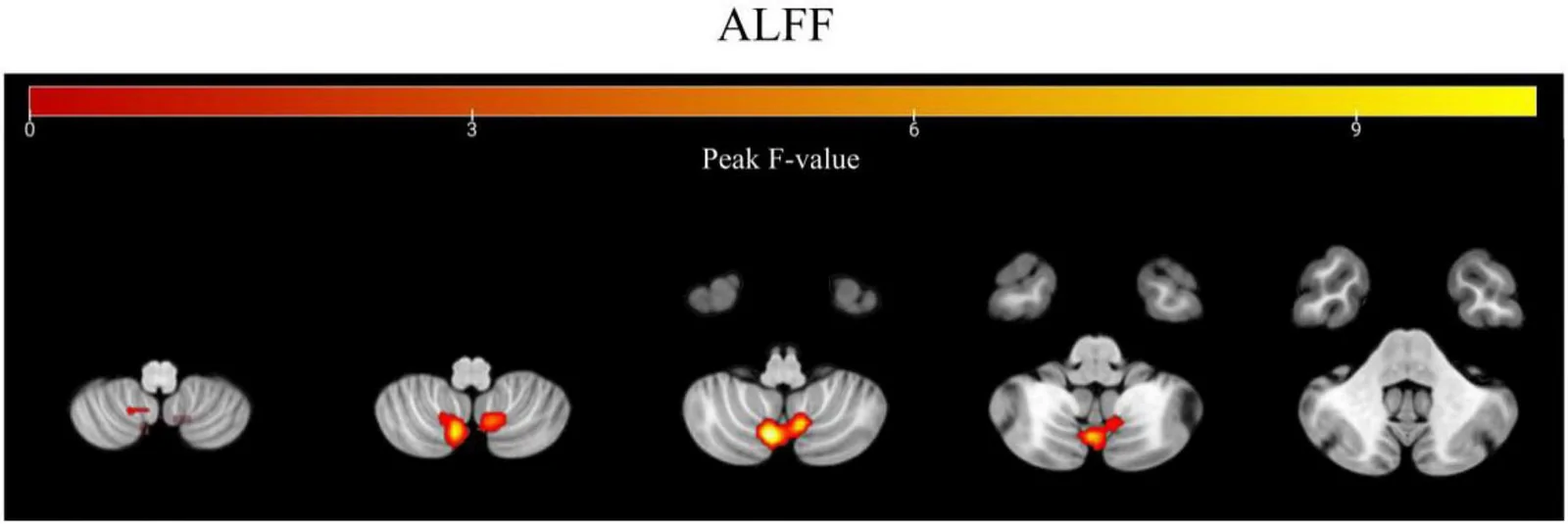
Brain regions showing significant differences in ALFF among the three groups defined by MMSE language-related task performance: intact performance, mildly reduced performance, and greater reduction in performance (ANCOVA).

**FIGURE 3:**
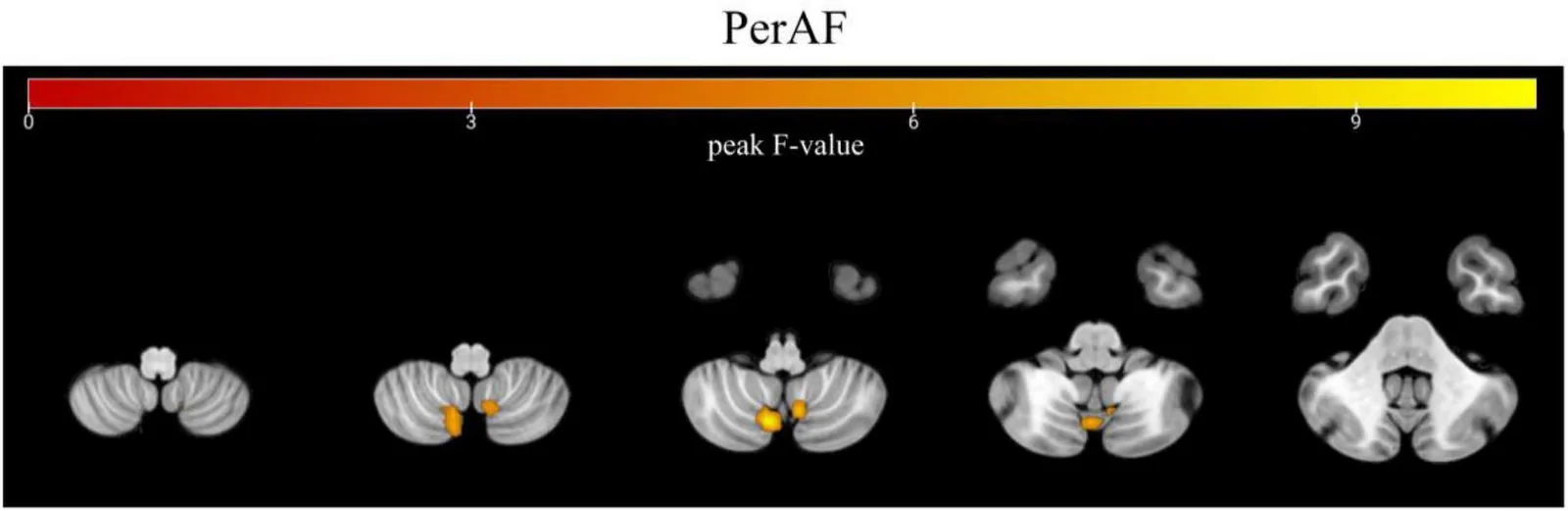
Brain regions showing significant differences in PerAF among the three groups defined by MMSE language-related task performance: intact performance, mildly reduced performance, and greater reduction in performance (ANCOVA).

The ALFF and PerAF group-difference maps are shown in Figures 2, 3, respectively, and the fALFF group-difference map is shown in Supplementary Figure S7. Notably, the right cerebellum 8 emerged as a region of convergent functional alteration across both mALFF and mPerAF metrics, characterized by a significant increase in neural activity as MMSE language-related task performance declined. This pattern of hyperactivity, alongside the increased occipital involvement in mfALFF, suggests a complex reorganization of neural circuits that may reflect compensatory recruitment or broader disease-related functional alterations.

#### 3.3.2 Frequency-specific alterations via wavelet-ALFF

To further investigate the frequency-specific patterns of neural oscillations, Wavelet-ALFF (W-ALFF) was analyzed across the conventional broadband (0.01–0.08 Hz), slow-5 (0.01–0.027 Hz), and slow-4 (0.027–0.073 Hz) bands. One-way ANCOVA revealed significant group differences in the right cerebellum 8 (Cerebellum_8_R) across both the conventional and slow-4 bands Table 8.

**TABLE 8:** Brain regions showing significant differences in frequency-specific alterations via wavelet-ALFF among the intact performance group, mildly reduced performance group, and greater reduction in performance group based on MMSE language-related task performance (ANCOVA).

			MNI coordinate (mm)			
Frequency band/regions	Cluster size	Peak *F*-value	*X*	*Y*	*Z*	*Post hoc*
Conventional broadband wavelet-ALFF (0.01–0.08 Hz)						
Cerebellum_8_R	111	9.88	9	−69	−48	Mildly reduced > Intact, Greater reduction > Intact
Slow-4 band wavelet-ALFF (0.027–0.073 Hz)						
Cerebellum_8_R	8	12.33	9	−69	−51	Mildly reduced > Intact, Greater reduction > Intact, Greater reduction > Mildly reduced

All results were corrected using Gaussian Random Field (GRF) theory (Conventional broadband (0.01–0.08 Hz): voxel-level *p* < 0.05, cluster-level *p* < 0.05, two-tailed; slow-4 band wavelet-ALFF (0.027–0.073 Hz): voxel-level *p* < 0.001, cluster-level *p* < 0.05, two-tailed).

Specifically, in the conventional broadband (voxel-level *p* < 0.05, cluster-level *p* < 0.05), a significant cluster was identified in the right cerebellum 8 (Peak *F* = 9.88, Cluster size = 111 voxels). *Post-hoc* analyses showed that both the Mildly Reduced Performance Group and the Greater Reduction in Performance Group exhibited significantly higher W-ALFF values compared with the Intact Performance Group (Mildly Reduced Performance > Intact Performance; Greater Reduction in Performance > Intact Performance).

Notably, whereas the broadband and ALFF/PerAF cerebellar clusters were detected at more liberal voxel-level thresholds, the slow-4 cerebellar cluster survived the most stringent threshold (voxel-level *p* < 0.001), exhibiting the highest peak intensity (*F* = 12.33). This frequency-specific robustness—rather than cluster extent—constitutes the strongest statistical evidence for cerebellar involvement and points specifically to the slow-4 band.

**FIGURE 4:**
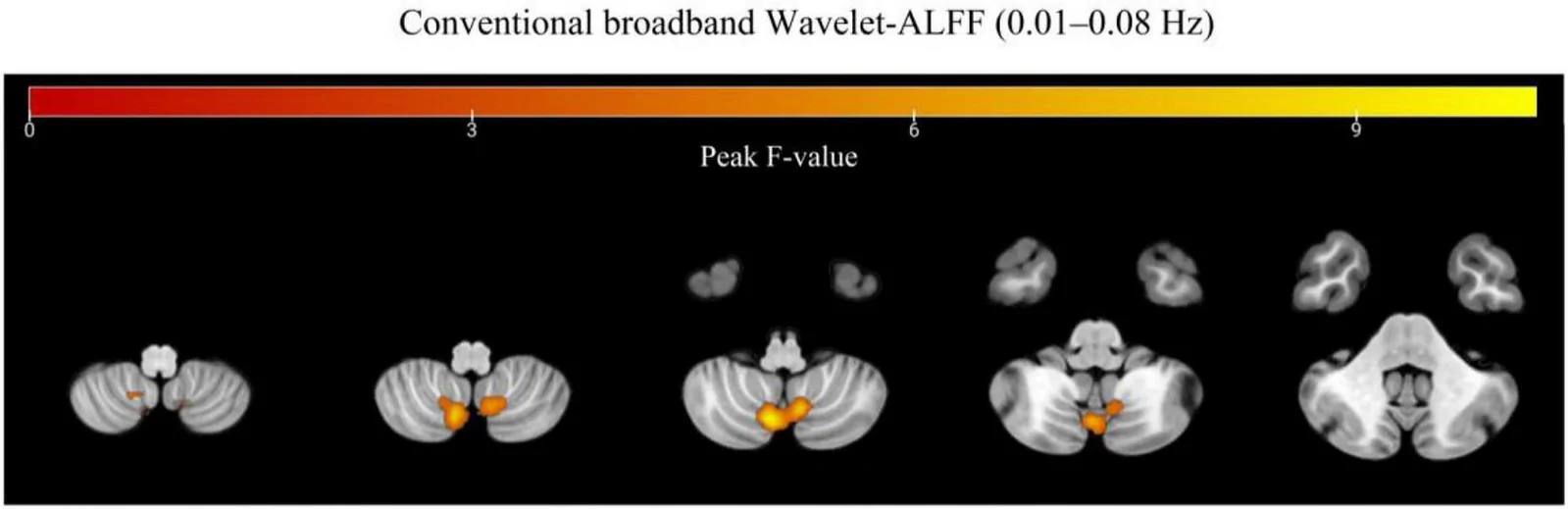
Brain regions showing significant differences in conventional broadband wavelet-ALFF (0.01–0.08 Hz) among the three groups defined by MMSE language-related task performance: intact performance, mildly reduced performance, and greater reduction in performance (ANCOVA).

**FIGURE 5:**
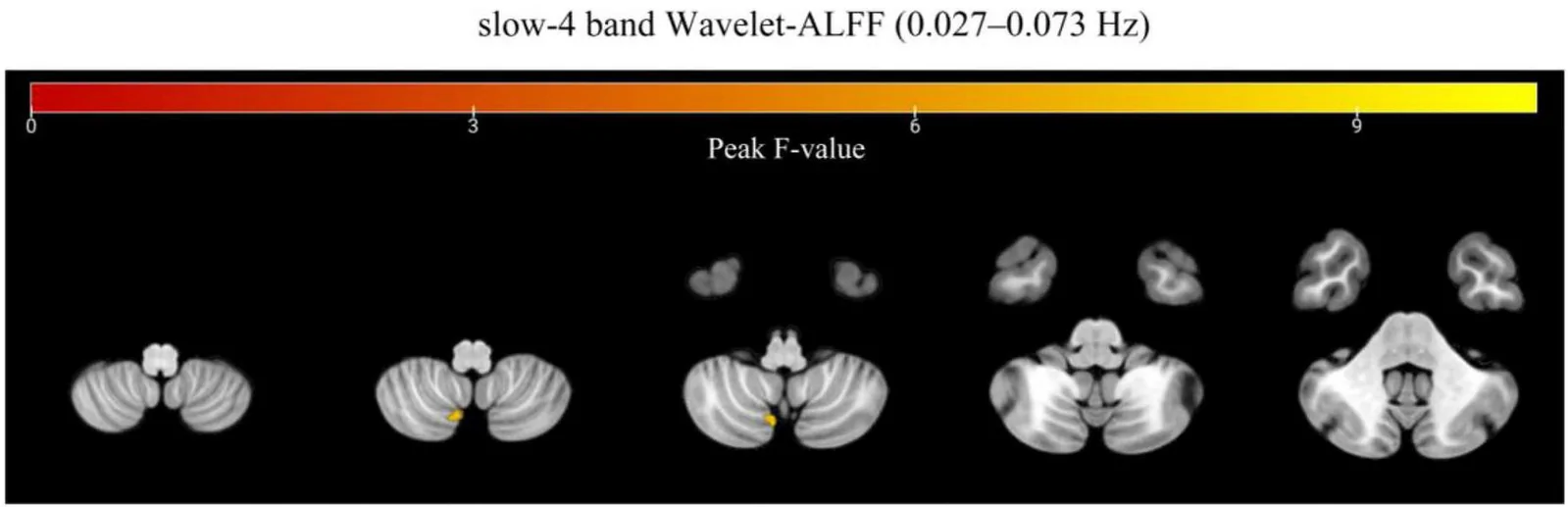
Brain regions showing significant differences in slow-4 band wavelet-ALFF (0.027–0.073 Hz) among the three groups defined by MMSE language-related task performance: intact performance, mildly reduced performance, and greater reduction in performance (ANCOVA).

The wavelet-ALFF group-difference maps are shown in Figures 4, 5. These findings indicate that the functional alterations in the right cerebellum 8 are frequency-dependent, with the slow-4 band demonstrating greater sensitivity to group-level differences in MMSE language-related task performance than the slow-5 and conventional broadband ranges. This frequency-specific manifestation suggests that the slow-4 oscillatory dynamics in the cerebellum may be particularly sensitive to variation in MMSE language-related task performance in this cohort.

### 3.4 Associations between rs-fMRI Metrics and MMSE language-related task performance

Voxel-wise multiple linear regression was performed to identify regions in which spontaneous neural activity covaried with MMSE language-related task performance. In each model, the voxel-wise rs-fMRI metric was entered as the dependent variable and the MMSE language sub-score as the predictor of interest, with age, sex, and years of education as covariates. A negative regression coefficient therefore indicates that higher spontaneous activity was associated with lower MMSE language-related task performance. Statistical significance was determined using Gaussian Random Field (GRF) theory (see Table 9 for metric-specific thresholds).

**TABLE 9:** Associations between rs-fMRI metrics and MMSE language-related task performance.^1^

			MNI coordinate (mm)		
Metric or frequency band/regions	Cluster size	*t*-value	X	Y	Z
ALFF					
Parietal_Sup_R	304	−3.34	24	−48	66
fALFF					
Lingual_R	11	−3.82	9	−81	−12
PerAF					
Postcentral_R	74	−3.79	27	−48	66
Conventional broadband wavelet-ALFF (0.01–0.08 Hz)					
Cerebellum_8_L	61	−3.92	6	−69	−48
Slow-5 band wavelet-ALFF (0.01–0.027 Hz)					
Cerebellum_8_R	21	−4.35	9	−69	−51
Parietal_Sup_L	19	−4.28	−30	−45	63
Slow-4 band wavelet-ALFF (0.027–0.073 Hz)					
Cerebellum_8_R	20	−4.21	9	−69	−51

^1^Clusters labeled as Cerebellum_8_L and Cerebellum_8_R in AAL parcellation were located near the midline (peak x = 6 and x = 9, respectively). Given the near-midline location of all three peaks, these clusters are interpreted collectively as paravermal (predominantly right) lobule VIII rather than as genuinely lateralized left/right effects. All results were corrected using Gaussian Random Field (GRF) theory (ALFF: voxel-level *p* < 0.05, cluster-level *p* < 0.05, two-tailed; fALFF: voxel-level *p* < 0.005, cluster-level *p* < 0.05, two-tailed; PerAF: voxel-level *p* < 0.001, cluster-level *p* < 0.05, two-tailed; Conventional broadband (0.01–0.08 Hz): voxel-level *p* < 0.05, cluster-level *p* < 0.05, two-tailed; slow-5 band wavelet-ALFF (0.01–0.027 Hz): voxel-level *p* < 0.001, cluster-level *p* < 0.05, two-tailed; slow-4 band wavelet-ALFF (0.027–0.073 Hz): voxel-level *p* < 0.001, cluster-level *p* < 0.05, two-tailed).

Specifically, mALFF in the right superior parietal gyrus was significantly negatively associated with MMSE language-related task performance (Peak = −3.34, Cluster size = 304 voxels). For mfALFF, a negative association was observed in the right lingual gyrus (Peak = −3.82, Cluster size = 11 voxels). For mPerAF, activity in the right postcentral gyrus was significantly negatively associated with MMSE language-related task performance (Peak = −3.79, Cluster size = 74 voxels).

Regarding wavelet-ALFF, frequency-specific negative associations were identified across different bands. In the conventional broadband (0.01–0.08 Hz), the left cerebellum 8 was negatively associated with language scores (Peak = −3.92, Cluster size = 61 voxels). In the slow-5 band (0.01–0.027 Hz), negative correlations were found in the right cerebellum 8 (Peak = −4.35, Cluster size = 21 voxels) and the left superior parietal gyrus (Peak = −4.28, Cluster size = 19 voxels). Similarly, in the slow-4 band (0.027–0.073 Hz), the right cerebellum 8 also demonstrated a significant negative correlation (Peak = −4.21, Cluster size = 20 voxels).

We note that, although the broadband regression cluster was labeled Cerebellum_8_L by the AAL atlas, its peak (*x* = 6) lay essentially on the midline, immediately adjacent to the slow-4 and slow-5 peaks (*x* = 9). Given the near-midline location of all three peaks and the limited resolution of atlas-based parcellation at the vermis, these clusters most plausibly reflect a single paravermal lobule VIII region straddling the midline rather than genuinely lateralized effects. We therefore describe the finding as involving paravermal (predominantly right) lobule VIII and refrain from strong lateralization claims.

**FIGURE 6:**
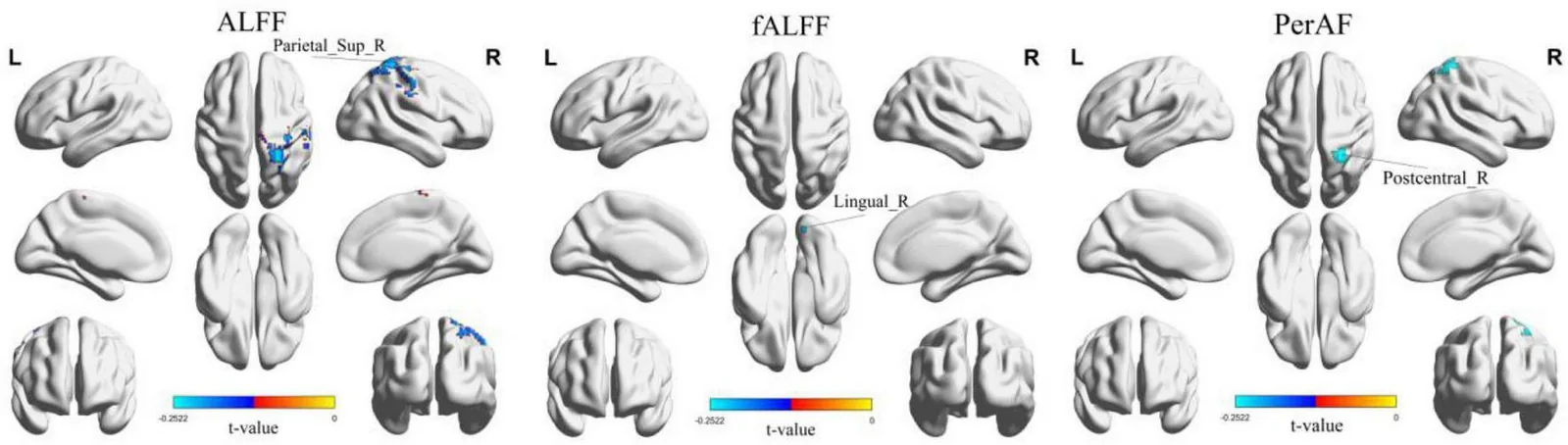
Brain regions showing significant negative associations of ALFF, fALFF, and PerAF with MMSE language-related task performance.

**FIGURE 7:**
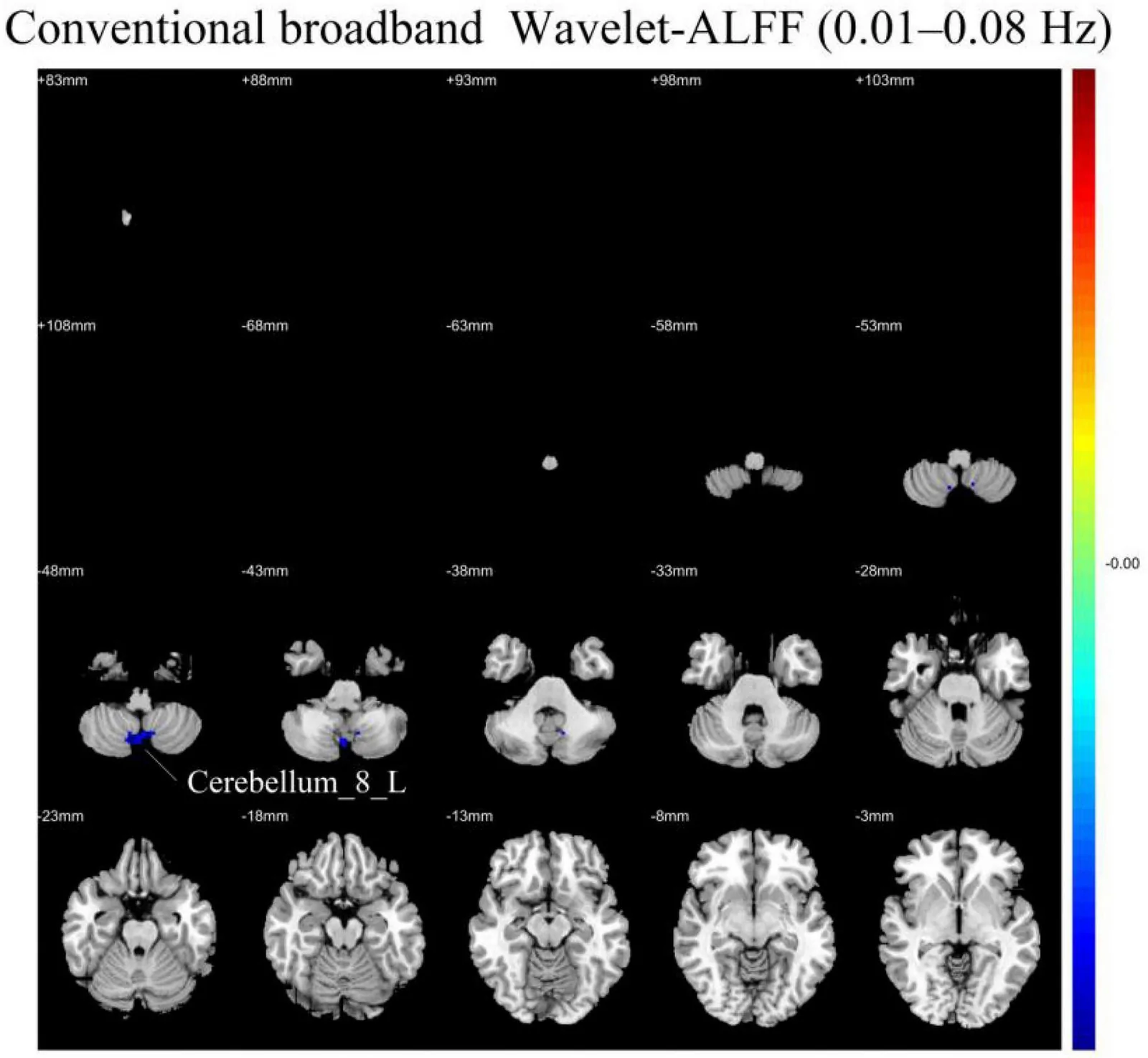
Brain regions showing significant negative associations between conventional broadband wavelet-ALFF (0.01–0.08 Hz) and MMSE language-related task performance.

**FIGURE 8:**
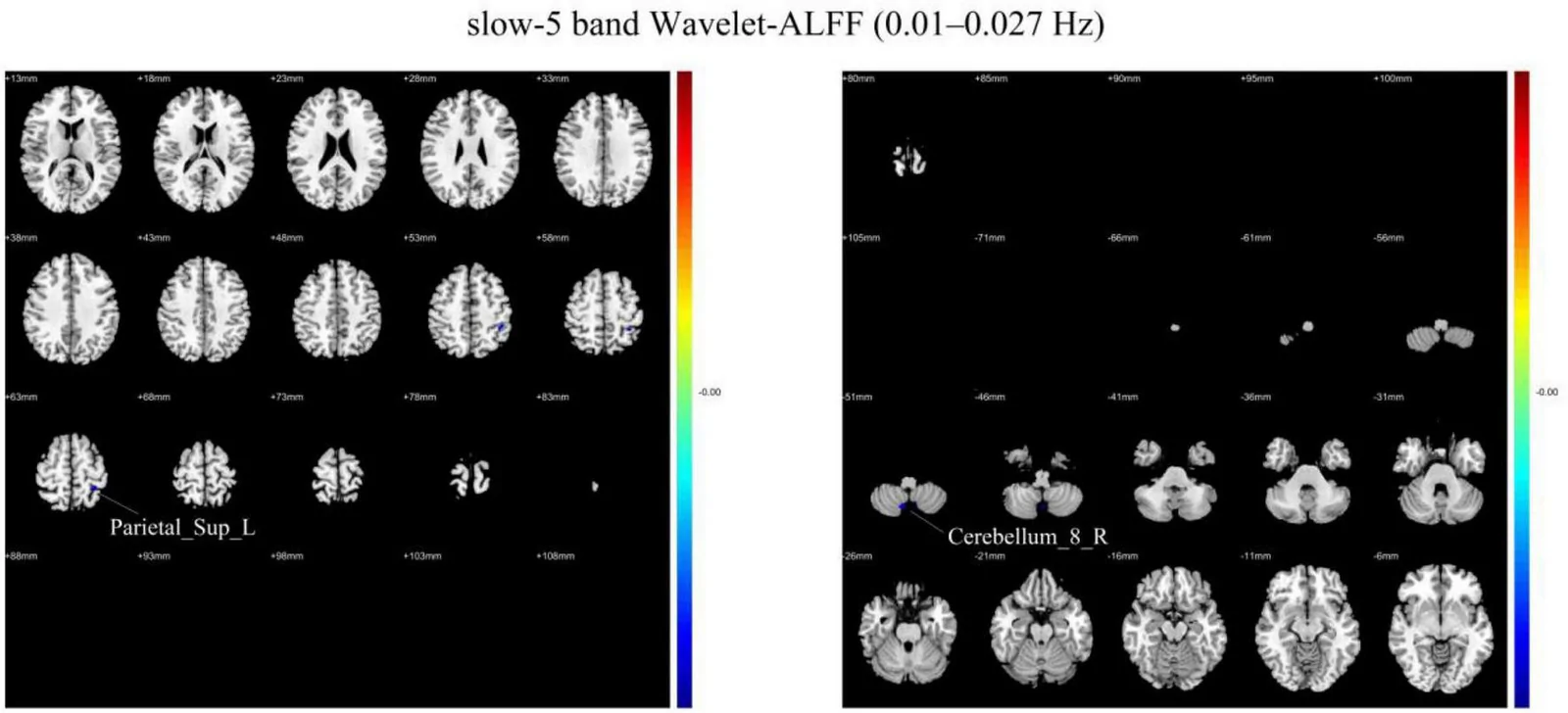
Brain regions showing significant negative associations between slow-5 band wavelet-ALFF (0.01–0.027 Hz) and MMSE language-related task performance.

**FIGURE 9:**
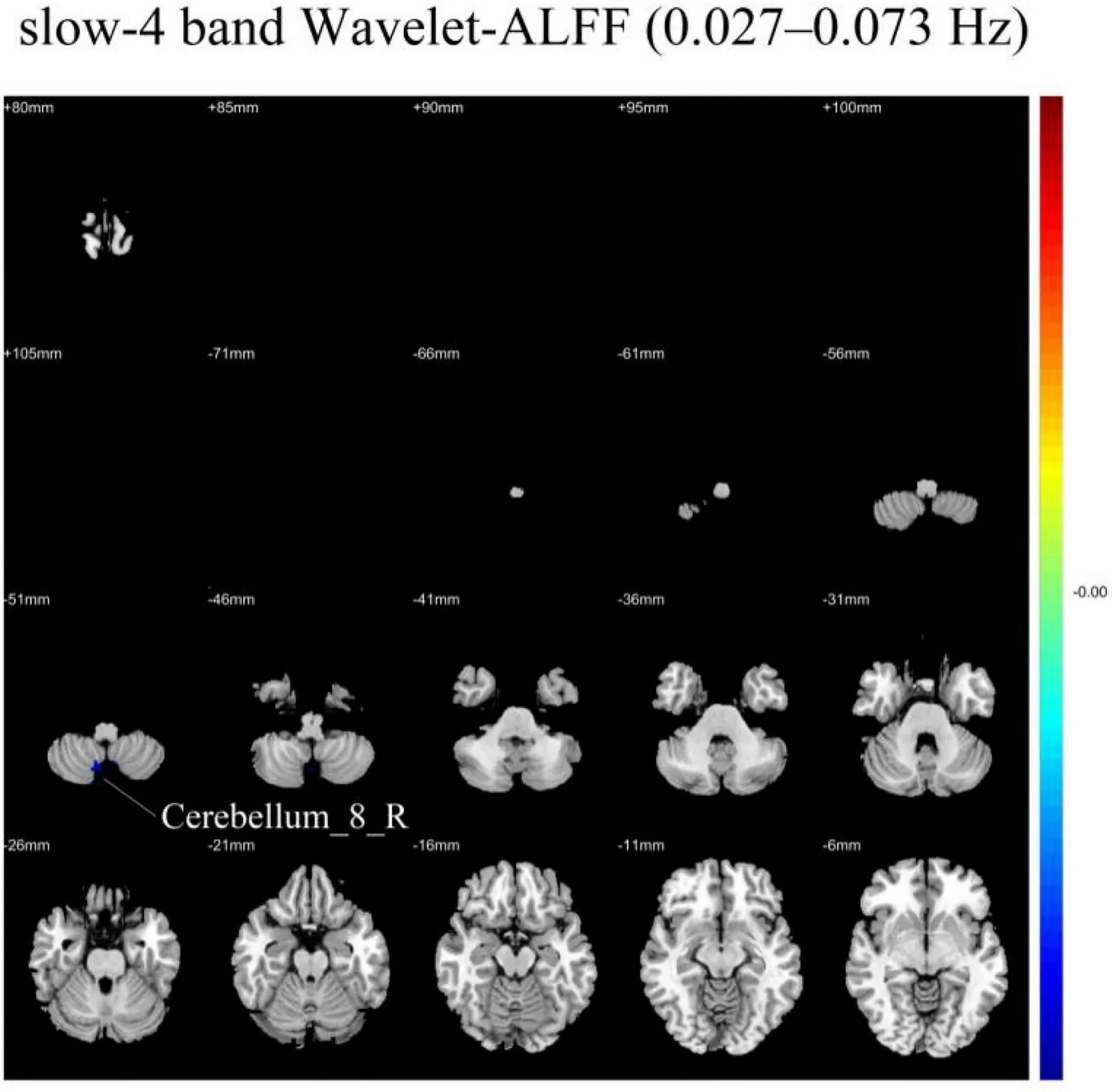
Brain regions showing significant negative associations between slow-4 band wavelet-ALFF (0.027–0.073 Hz) and MMSE language-related task performance.

The correlation maps are shown in Figures 6–9. These results indicate that increased spontaneous neural activity in the parietal, occipital, and cerebellar regions is associated with poorer MMSE language-related task performance. The consistent involvement of paravermal lobule VIII across multiple frequency bands suggests that cerebellar intrinsic activity is associated with individual differences in MMSE language-related task performance across the AD spectrum, although the present data cannot specify the underlying mechanism. Importantly, language scores were additionally modeled as a continuous variable in the voxel-wise regression analyses to evaluate the robustness of the categorical ANCOVA findings. The continuous analyses yielded a pattern broadly consistent with the group-wise comparisons, with right cerebellar lobule VIII emerging as one of the most consistently implicated regions across metrics and frequency bands. The convergence between categorical and continuous analytical approaches therefore increases confidence that the observed cerebellar findings were not solely dependent on a particular grouping strategy.

**FIGURE 10:**
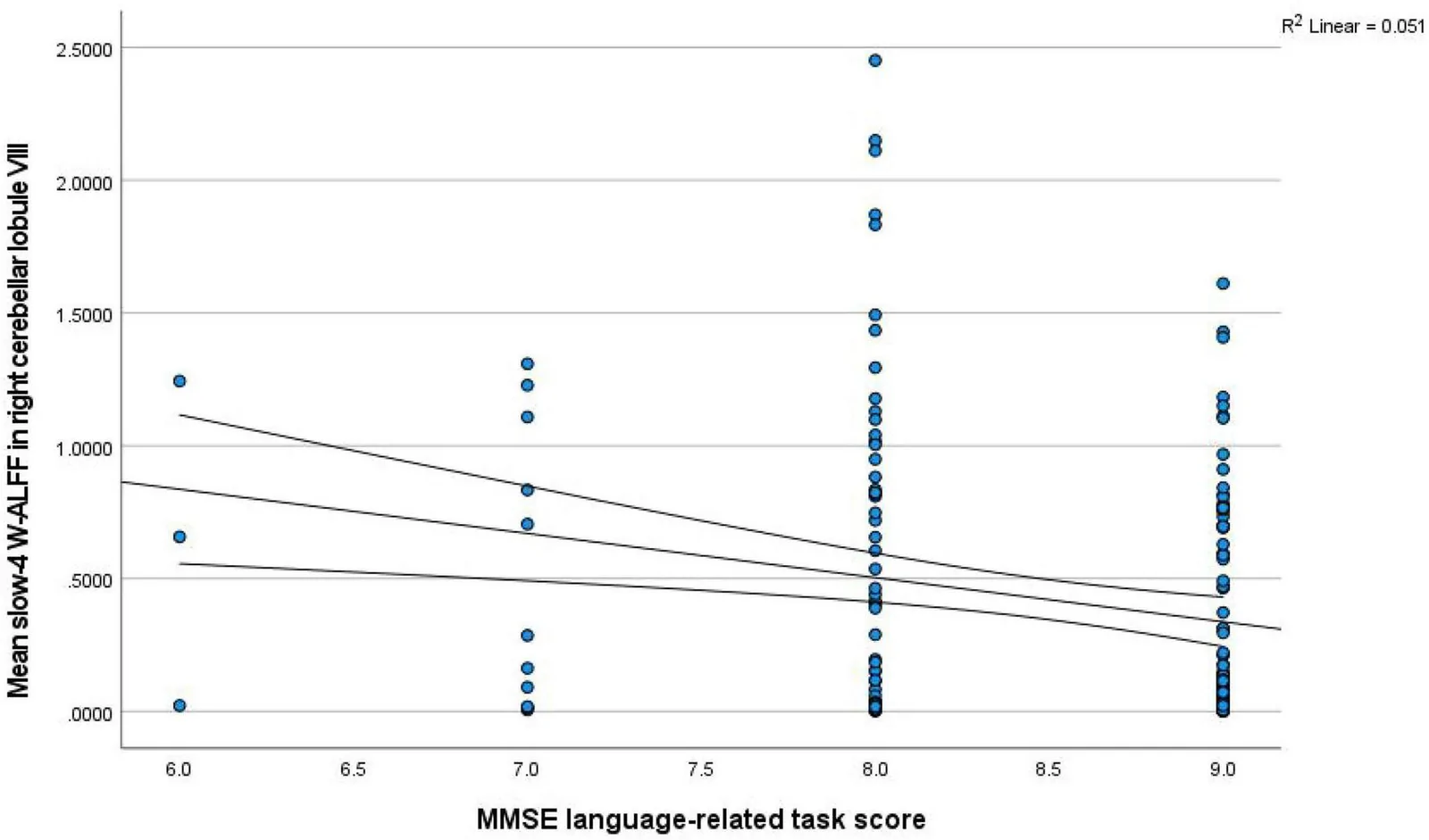
ROI-based visualization of the association between mean slow-4 wavelet-ALFF values in right cerebellar lobule VIII and MMSE language-related task performance.

To improve the interpretability of the cerebellar finding, we extracted the mean slow-4 Wavelet-ALFF values from the significant right cerebellar lobule VIII ROI identified in the voxel-wise analysis. As illustrated in Figure 10, higher slow-4 activity in this cerebellar region was visually associated with lower MMSE language-related task scores. This ROI-based scatterplot was included for visualization of the main voxel-wise association and should not be interpreted as an independent confirmatory analysis.

### 3.5 ROI-Based sensitivity analyses controlling for global cognitive severity

To examine whether the association between right cerebellar lobule VIII activity and MMSE language-related task performance was driven by global cognitive impairment or overall disease severity, we conducted additional ROI-based sensitivity analyses. Mean slow-4 wavelet-ALFF values were extracted from the significant right cerebellar lobule VIII cluster identified in the main analysis.

Partial correlation analyses were then performed between the extracted ROI values and MMSE language subscores, with additional adjustment for global cognitive measures. Because the MMSE total score includes the MMSE language subscore, directly controlling for the MMSE total score would introduce construct overlap. Therefore, two complementary models were used. Model A included age, sex, education, and CDR-SB as covariates to account for global clinical disease severity. Model B included age, sex, education, and the MMSE non-language score, calculated as MMSE total score minus the MMSE language subscore, to account for broader non-language cognitive performance. The ROI-based sensitivity analyses are summarized in Table 10.

**TABLE 10:** ROI-based sensitivity analyses for the association between slow-4 wavelet-ALFF in right cerebellar lobule VIII and MMSE language-related task performance.

Model	Covariates	Partial r	*df*	*p*-value
Model A	Age, sex, education, CDR-SB	−0.306	169	*p* < 0.001
Model B	Age, sex, education, MMSE non-language score	−0.314	169	*p* < 0.001

The negative association between slow-4 wavelet-ALFF in right cerebellar lobule VIII and MMSE language-related task performance remained significant after adjusting for CDR-SB (*r* = −0.306, df = 169, *p* < 0.001). This association also remained significant after adjusting for the MMSE non-language score (*r* = −0.314, df = 169, *p* < 0.001). These findings suggest that the observed cerebellar association was not fully explained by global clinical disease severity or non-language MMSE performance.

Because the ROI was defined from the same sample in which the voxel-wise effect was identified, these sensitivity analyses are not statistically independent of the primary finding; they are intended to test the specificity of the association against global severity rather than to provide independent confirmation. The consistency of the effect size before and after adjustment for CDR-SB and non-language MMSE performance nonetheless argues against the cerebellar association being a mere proxy for global decline.

### 3.6 Summary of key findings

Three principal findings emerged from the present analyses. First, intrinsic brain activity across the AD spectrum showed frequency-dependent spatial patterns: temporal regions were more prominently affected in the slow-5 band, whereas parietal regions were more involved in the slow-4 band. Second, paravermal (predominantly right) lobule VIII was consistently identified across multiple local amplitude metrics, including ALFF, PerAF, and wavelet-ALFF, as well as across categorical and continuous analyses of MMSE language-related task performance. This involvement was frequency-sensitive, with the most consistent cross-analytic support observed in the slow-4 band. Critically, among the cerebellar clusters identified in the categorical group comparisons, only the slow-4 cluster survived the most stringent voxel-level threshold (*p* < 0.001; *F* = 12.33). The slow-4 cerebellar finding also remained significant in the continuous regression analysis at the same voxel-level threshold. This frequency-specific statistical robustness—rather than cluster extent or metric selection—provides the strongest evidence that the slow-4 band is specifically linked to MMSE language-related task performance. Third, increased spontaneous activity in right cerebellar lobule VIII was negatively associated with MMSE language-related task performance. Importantly, this association remained significant in ROI-based sensitivity analyses after adjustment for CDR-SB and after adjustment for the MMSE non-language score. These results suggest that the cerebellar association was not fully attributable to global clinical disease severity or broader non-language cognitive performance. Taken together, the findings identify right cerebellar lobule VIII as a robust correlate of MMSE language-related task performance across the AD spectrum, while also underscoring the need to interpret this association cautiously given the composite nature of the MMSE language subscore.

## 4 Discussion

The present study employed a multi-metric, frequency-resolved resting-state fMRI framework to investigate alterations in spontaneous neural activity across the Alzheimer’s disease (AD) spectrum and their associations with performance on MMSE language-related tasks. By integrating ALFF, fALFF, PerAF, and wavelet-ALFF within the slow-4 and slow-5 frequency bands, we addressed three interrelated questions: (1) whether intrinsic brain activity measures differ across diagnostic stages of AD; (2) whether these alterations are frequency-dependent; and (3) which local amplitude alterations are associated with individual differences in MMSE language-related task performance. Our analyses yielded three principal findings. First, amplitude-based metrics revealed convergent and complementary patterns of intrinsic activity alterations across the AD continuum. Second, these alterations exhibited frequency-dependent topography, with temporal regions more prominent in the slow-5 band and parietal regions—particularly the right angular gyrus—more involved in the slow-4 band. Third, right cerebellar lobule VIII was consistently identified across multiple metrics, frequency bands, and analytical approaches. Importantly, this finding should be interpreted as a brain–behavior association with a brief composite screening measure, rather than as direct evidence for a language-specific cerebellar mechanism.

The most informative result of this study is not the cerebellar finding in isolation, but the contrast between two analytic anchors. When intrinsic activity was compared across diagnostic stages, the differentiating regions were cortical, including supramarginal, angular, frontal, and temporal regions. Analyses anchored to MMSE language-related task performance also identified several cortical regions but repeatedly converged on paravermal (predominantly right) cerebellar lobule VIII across ALFF, PerAF, and wavelet-ALFF and across categorical and continuous models. This contrast between diagnosis-differentiating cortical patterns and the recurrent performance-related cerebellar finding constitutes the central analytic contribution of the study. Critically, this performance-anchored cerebellar association persisted after adjustment for CDR-SB and for non-language MMSE performance, indicating that it does not simply re-index diagnostic stage or global severity. This contrast demonstrates that, in large phenotype-anchored neuroimaging datasets, the regions that best separate clinical stages are not necessarily those that best track a specific behavioral measure (Jack et al., 2013). Methodologically, it also illustrates the added value of a frequency-resolved, multi-metric framework: had we relied on a single metric or a single frequency band, the cerebellar signal—weakest in cluster extent but strongest in frequency specificity—might well have been missed or dismissed as noise. The convergence of a modest effect across metrics, frequency bands, and modeling strategies reduces concern that the finding is driven by any single thresholding choice, although independent replication remains necessary.

In cross-diagnostic group comparisons, ALFF highlighted the right supramarginal gyrus, PerAF identified the left inferior temporal gyrus, whereas fALFF identified broader frontotemporal alterations. This partial convergence and divergence suggests that different spontaneous activity indices may capture overlapping but non-identical aspects of intrinsic neural dynamics (Zang et al., 2007; Zou et al., 2008). ALFF indexes the amplitude of low-frequency fluctuations, whereas PerAF standardizes signal fluctuations relative to voxel-wise mean signal intensity, improving inter-subject comparability and test–retest reliability in multi-center datasets (Jia et al., 2020; Hammers et al., 2022). fALFF, by normalizing low-frequency power against the full detectable frequency spectrum, reduces physiological noise and may be more sensitive to shifts in relative spectral power distribution (Zou et al., 2008; Zuo et al., 2010). The right supramarginal gyrus has been implicated in phonological processing and multimodal integration (Binder et al., 2009; Hartwigsen et al., 2010). However, because the present analyses were based on local amplitude metrics, these regional findings should be understood as markers of altered intrinsic activity rather than as evidence for specific functional pathways or causal mechanisms.

A central contribution of this study lies in demonstrating that AD-related intrinsic activity alterations exhibit frequency-dependent spatial patterns. Wavelet decomposition revealed that slow-5 band changes were more prominent in temporal regions, whereas slow-4 band alterations were concentrated in parietal hubs, particularly the right angular gyrus. Prior work has suggested that sub-bands within the conventional low-frequency range may index partially distinct physiological processes and network characteristics (Penttonen and Buzsáki, 2003; Yue et al., 2023). Slow-5 oscillations have been linked to cortical slow waves and default-mode network integration, whereas slow-4 oscillations have been considered more sensitive to cortico-subcortical information transfer (Snyder, 2022; Yue et al., 2023). The preferential involvement of temporal regions in the slow-5 band is broadly consistent with the vulnerability of memory–semantic systems in early AD (Verma and Howard, 2012). Conversely, slow-4 alterations in angular and supramarginal regions—areas implicated in multimodal semantic convergence—may be consistent with altered large-scale functional dynamics in AD, although the present study did not directly test functional connectivity or cortico-subcortical coupling (Binder et al., 2009). Thus, frequency-resolved analysis may enhance sensitivity to regionally specific alterations, but the physiological mechanisms underlying these frequency-dependent patterns require further validation.

What might this performance-anchored cerebellar signal reflect? We deliberately refrain from asserting a specific mechanism, because local resting-state amplitude metrics cannot adjudicate among connectivity-, network-, or task-level accounts. Nonetheless, two features of the finding constrain plausible interpretations. First, lobule VIII is consistently mapped to somatomotor representations in cerebellar functional parcellations (Buckner et al., 2011; Yeo et al., 2011), and the MMSE language-related items—naming, repetition, reading, writing, command execution, and constructional praxis—all impose articulatory, motor-planning, and visuomotor demands. The association may therefore be driven, at least in part, by the sensorimotor and execution components embedded in these tasks rather than by linguistic processing *per se*. Second, the direction of the effect is noteworthy: greater spontaneous cerebellar activity accompanied poorer performance. Rather than a simple loss-of-function pattern, this inverse relationship may be consistent with inefficient or dysregulated cerebellar recruitment in the context of progressive neurodegeneration (Gollo et al., 2017), although a compensatory interpretation cannot be distinguished from a maladaptive one with the present cross-sectional, amplitude-based data. These accounts are offered as hypotheses to be tested with task-based, connectivity, and longitudinal designs, not as conclusions.

Beyond these task-related considerations, the association may also relate to broader disease-associated alterations in cerebello-thalamo-cortical systems as neurodegenerative burden accumulates (Schmahmann and Sherman, 1998; Thal et al., 2002). None of these possibilities, however, can be adjudicated by the present data, which include neither task-based fMRI, functional or effective connectivity, diffusion MRI, nor longitudinal modeling.

The frequency-specific nature of the cerebellar finding also warrants cautious interpretation. Previous studies have suggested that slow-4 oscillations may be sensitive to cortico-subcortical and cerebro-cerebellar interactions (Yue et al., 2023). In the present study, right cerebellar lobule VIII showed its most consistent cross-analytic support in the slow-4 band. This pattern may be consistent with frequency-specific involvement of cerebellar systems in AD-related cognitive and behavioral changes. However, because the present analyses were based exclusively on local resting-state amplitude metrics, they cannot directly test cortico-cerebellar coupling, effective connectivity, white-matter pathways, or task-evoked cerebellar recruitment. Future studies combining frequency-resolved resting-state analyses with connectivity and task-based approaches will be necessary to clarify the functional significance of this slow-4 cerebellar association.

Another important interpretational issue concerns global cognitive severity. The primary voxel-wise analyses controlled for age, sex, and education but did not include measures of global cognitive severity. However, the subsequent ROI-based sensitivity analyses additionally adjusted for CDR-SB and MMSE non-language performance, and the negative association between slow-4 wavelet-ALFF in paravermal (predominantly right) cerebellar lobule VIII and MMSE language-related task performance remained significant in both models. These findings suggest that the observed association was not fully explained by global clinical disease severity or broader non-language cognitive performance. Nevertheless, because the ROI was defined from the same sample in which the voxel-wise effect was identified, these sensitivity analyses were not statistically independent and should be interpreted as specificity analyses rather than as independent confirmation. Future studies should replicate this association using independently defined ROIs and more comprehensive measures of language and global cognition.

This diagnosis-versus-performance analytic contrast is methodologically important for phenotype-anchored analyses of large datasets: regions that best separate diagnostic stages need not coincide with those that track a specific behavioral measure, and conflating the two can misattribute behavioral correlates to disease staging (Jack et al., 2013).

Finally, the translational implications of the present findings should be interpreted with substantial caution. The current study was cross-sectional, observational, and correlational, and therefore cannot determine whether altered activity in right cerebellar lobule VIII represents a primary pathological process, a compensatory response, or a secondary consequence of broader neurodegeneration. Accordingly, the present findings do not establish cerebellar lobule VIII as a therapeutic target, nor do they support the conclusion that cerebellar neuromodulation would improve language-related performance in AD or MCI. Rather, the observed association should be regarded as hypothesis-generating. Future longitudinal and interventional studies will be needed to determine whether cerebellar activity is causally related to cognitive, sensorimotor, or MMSE language-related task performance across the AD spectrum. In this context, non-invasive neuromodulation approaches such as repetitive transcranial magnetic stimulation or transcranial direct current stimulation may provide experimental tools for testing causal hypotheses in future work, but such applications require direct validation in appropriately designed intervention studies (Bonnì et al., 2014; Dubovik et al., 2013). Emerging histopathological and neuroimaging evidence suggests that cerebellar involvement may occur in certain stages or variants of AD (Xie et al., 2024; Katsuse et al., 2025). The present findings suggest that cerebellar circuits warrant further investigation in studies designed to test causal mechanisms.

Several limitations should be emphasized. First, the MMSE language subscore is a brief composite screening measure and cannot be considered a pure measure of language function. Its component tasks involve linguistic, executive, visuospatial, motor, and sensorimotor processes. Second, the study relied on local resting-state amplitude metrics and did not include task-based activation, functional connectivity, effective connectivity, structural connectivity, or network-level modeling. As a result, the present findings cannot directly establish sensorimotor integration, phonological-loop dysfunction, forward-model processing, motor-semantic integration, or cortico-cerebellar coupling abnormalities. Third, global cognitive severity was not included in the primary voxel-wise analyses. Although the ROI-based sensitivity analyses additionally adjusted for CDR-SB and MMSE non-language performance and the associations remained significant, the ROI was defined from the same sample; therefore, these analyses were not statistically independent and require independent replication. Fourth, the cross-sectional design precludes causal inference. Fifth, molecular biomarkers such as amyloid and tau measures were not incorporated into the present analyses. Finally, the MMSE language-related task performance groups were markedly imbalanced, with a relatively small number of participants in the greater reduction group, which may affect the stability of categorical group comparisons.

In summary, this study demonstrates that frequency-resolved local resting-state amplitude metrics reveal spatially and spectrally distinct alterations across the AD spectrum. Right cerebellar lobule VIII emerged as a consistent correlate of MMSE language-related task performance, particularly in the slow-4 band. The findings suggest that cerebellar intrinsic activity is associated with variation in performance on a composite MMSE language-related screening measure, but they do not provide direct evidence for a language-specific or mechanistic cerebellar account. Future studies using comprehensive language batteries, global cognitive covariates, task-based fMRI, functional and effective connectivity, diffusion MRI, molecular biomarkers, and longitudinal designs will be essential for clarifying whether and how cerebellar systems contribute to language-related, cognitive, and sensorimotor changes across the AD spectrum.

## 5 Conclusion

This study investigated frequency-specific alterations in spontaneous neural activity associated with MMSE language-related task performance across the Alzheimer’s disease spectrum using a multi-metric resting-state fMRI framework. The findings revealed frequency-dependent spatial patterns of intrinsic activity alterations, with temporal regions more prominently involved in the slow-5 band and parietal regions, particularly the right angular gyrus, more prominently involved in the slow-4 band. Across multiple amplitude metrics and analytical approaches, paravermal (predominantly right) lobule VIII emerged as a consistent correlate of MMSE language-related task performance, especially within the slow-4 frequency range. ROI-based sensitivity analyses further showed that this association remained significant after controlling for CDR-SB and MMSE non-language performance, indicating that it was not fully explained by global cognitive severity. These findings should be interpreted cautiously given the cross-sectional and correlational nature of the study, the use of a composite screening measure rather than a comprehensive language battery, and the imbalance in language-group sample sizes, all of which are fully discussed in the Discussion. Nevertheless, the convergence of evidence across multiple metrics, frequency bands, and analytic strategies—including both categorical and continuous approaches—supports the validity of the cerebellar association and partially mitigates concerns about group imbalance. The present results provide a hypothesis-generating foundation for future longitudinal and interventional studies to examine whether cerebellar circuits play a causal role in cognitive and sensorimotor changes across the AD spectrum, and to clarify their potential relevance for mechanistic research.

## Data Availability

The datasets analyzed in this study are available from the Alzheimer’s Disease Neuroimaging Initiative (ADNI) database (https://adni.loni.usc.edu/) upon registration and approval.

## Author’s note

Data used in preparation of this article were obtained from the Alzheimer’s Disease Neuroimaging Initiative (ADNI) database (adni.loni.usc.edu). As such, the investigators within the ADNI contributed to the design and implementation of ADNI and/or provided data but did not participate in analysis or writing of this report. A complete listing of ADNI investigators can be found at: https://adni.loni.usc.edu/wp-content/uploads/how_to_apply/ADNI_Acknowledgement_List.pdf.
