# Supplementary material for: Slow-4 cerebellar activity tracks MMSE language-related task performance across the Alzheimer’s disease spectrum: a multi-metric resting-state fMRI study
Source: Front Neurosci. 2026 Aug 28;20:1820150. doi: 10.3389/fnins.2026.1820150 (PMC13563651; doi:10.3389/fnins.2026.1820150)
Supplement: Supplementary file 1 [file Supplementary_file_1.docx]

**Supplementary**

**Fig S1 Brain regions significant differences in ALFF among AD, MCI, and CN groups (Colored voxels indicate the suprathreshold ANCOVA F-map; peak F = 10.94)**

**Fig S2 Brain regions significant differences in fALFF among AD, MCI, and CN groups (ANCOVA)**

**Fig S3 Brain regions significant differences in PerAF among AD, MCI, and CN groups (ANCOVA)**

**Fig S4 Brain regions significant differences in Conventional broadband Wavelet-ALFF (0.01–0.08 Hz) among AD, MCI, and CN groups**

**(Colored voxels indicate the suprathreshold ANCOVA F-map; peak F = 11.44)**

**Fig S5 Brain regions showing significant differences in slow-5 band wavelet-ALFF (0.01–0.027 Hz) among the AD, MCI, and CN groups (ANCOVA)**

**Fig S6 Brain regions showing significant differences in slow-4 band wavelet-ALFF (0.027–0.073 Hz) among the AD, MCI, and CN groups (ANCOVA)**

**Fig S7 Brain regions showing significant differences in fALFF among the groups with intact performance, mildly reduced performance, and greater reduction in performance based on MMSE language-related task scores (ANCOVA)**
